# The Use of Edible Insect Species *Tenebrio molitor* (Mealworm), *Acheta domesticus* (House Cricket), and *Hermetia illucens* (Black Soldier Fly) in the Food Industry: Nutritional Value, Safety Profile, and Consumer Acceptance

**DOI:** 10.1007/s13668-026-00770-4

**Published:** 2026-05-22

**Authors:** Simge Cankaya, Hande Mortaş

**Affiliations:** 1 Institute of Health Sciences, Department of Nutrition and Dietetics, Gazi University, Ankara, 06490 Türkiye; 2 Faculty of Health Sciences, Department of Nutrition and Dietetics, Gazi University, Ankara, 06490 Türkiye

**Keywords:** Edible insects (entomophagy), Alternative protein sources, Protein quality (DIAAS/PDCAAS), Bioavailability, Processing technologies, Food safety, Consumer acceptance

## Abstract

**Purpose of Review:**

This review aims to explore the role of edible insects as an alternative protein source in the food industry, focusing on their nutritional value, processing feasibility, food safety, and consumer acceptance. It provides an integrative perspective on how edible insects can contribute to sustainable food systems while addressing challenges related to consumer perception and regulatory frameworks.

**Recent Findings:**

Recent studies have demonstrated that key edible insect species such as *Tenebrio molitor*, *Acheta domesticus*, and *Hermetia illucens L.* exhibit high protein content, favorable amino acid profiles, optimal omega-3/6 fatty acid ratios, and excellent bioavailability. Processing techniques including drying, extrusion, fermentation, and isolated protein production have been shown to influence both nutritional quality and microbiological safety. Moreover, consumer acceptance studies highlight that attitudes toward insect consumption are shaped by perceived benefits, disgust sensitivity, ethical considerations, and cultural familiarity.

**Summary:**

Edible insects present a promising alternative protein source with significant nutritional and environmental advantages. However, large-scale industrial adoption requires the combined development of scientific evidence, standardized regulations, and effective consumer education strategies. By integrating nutritional, technological, and psychosocial dimensions, this review provides a comprehensive understanding of the potential of edible insects in advancing sustainable food systems.

**Graphical Abstract: Production–processing–product development process of edible insects.:**

**Figure:**
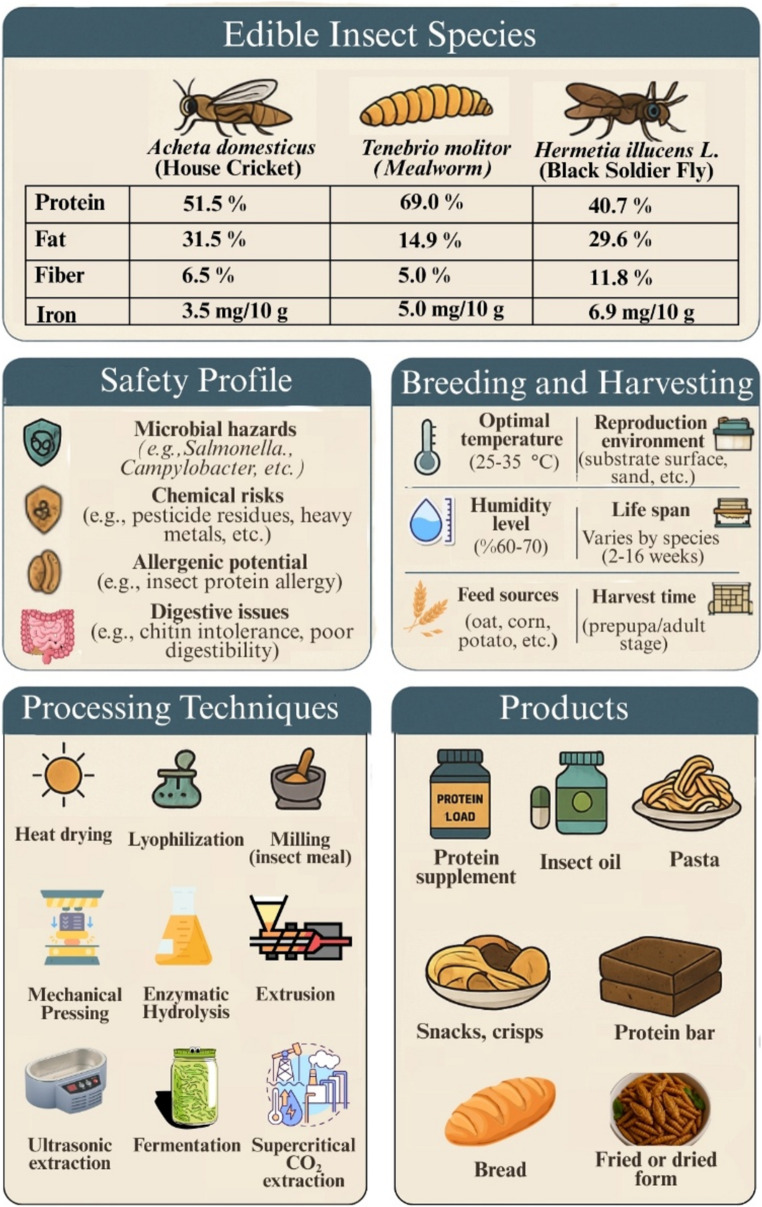
The Graphical Abstract illustrates three key edible insect species (*Acheta domesticus*, *Tenebrio molitor*, and *Hermetia illucens*) with their nutritional composition (protein, fat, fiber, and iron contents). The safety profile highlights microbial, chemical, allergenic, and digestive risks. Breeding and harvesting requirements include optimal temperature (25–35 °C), humidity level (60–70%), feed sources (oat, corn, potato), reproduction environment, life span, and harvest time. Processing techniques are presented sequentially, where each icon represents a step: heat drying, lyophilization, milling, mechanical pressing, enzymatic hydrolysis, extrusion, ultrasonic extraction, fermentation, and supercritical CO₂ extraction. The final icons show food products derived from insect biomass, such as protein supplements, insect oil, pasta, snacks, crisps, protein bars, bread, and fried/dried forms.

## Introduction

The rapid growth of the global population and ongoing urbanization have driven a qualitative shift in protein demand, with increasing interest in healthier, more sustainable, and ethically produced protein sources [1, 2]. Conventional animal-based food production systems intensify environmental burdens and pose various health and food safety challenges [3] .In this context, sustainable nutrition has gained importance for both individual health and the planet’s ecological balance. The Food and Agriculture Organization (FAO) and the World Health Organization (WHO) define sustainable nutrition as dietary patterns that support the health of all individuals, have low environmental impact, are accessible, affordable, safe, and culturally acceptable [4].

The livestock sector accounts for approximately 14.5% of global greenhouse gas emissions [5]​. The primary sources of these emissions include enteric fermentation, manure management, land conversion, and feed production [6]​. Moreover, livestock contributes to ecosystem degradation through water use, deforestation, biodiversity loss, and soil erosion [7]​. In contrast, alternative protein sources offer sustainable and innovative options that can substitute for conventional animal proteins [2, 8, 9]. These sources are generally classified into four main groups: plant-based proteins, microbial proteins (algae and fungi), insect-based proteins, and cultured (lab-grown) meat [10, 11]. Assessing these alternative protein sources requires consideration of multidimensional parameters such as protein content, digestibility, essential amino acid composition, sensory characteristics, and technological functionality. For instance, the larva of the mealworm (*Tenebrio molitor*) exhibits a superior profile with approximately 25% protein concentration compared to conventional red meat products containing 15–20% protein (Orkusz, 2021). Conversely, studies on cultured meat products produced via biotechnological methods in vitro remain limited, particularly concerning protein bioavailability and digestibility (Tavan et al., 2025). On the other hand, plant-based meat analogs are structured using protein aggregates to mimic the texture and organoleptic qualities of traditional meat (He et al., 2020). However, the biological value and post-digestion amino acid absorption rates of plant-based proteins are often lower than those of animal proteins due to limited profiles of essential amino acids, especially lysine, methionine, and tryptophan (Zhang et al., 2021). Such disadvantages are partially compensated through processing technologies such as extrusion, fermentation, and enzymatic modification, while their functional properties—including texturization, water-holding capacity, and emulsification—play significant roles in product formulation. Additionally, the acceptance of plant-based proteins varies across regions, influenced by consumer perception, taste profile, and perceived naturalness [2, 10, 12, 13]. In developing countries, animal protein is often regarded as a symbol of prosperity, which may limit the shift toward plant-based alternatives [14]. Limited studies conducted in Türkiye indicate a general curiosity toward plant-based meat products, yet factors such as price, taste, and familiarity significantly affect consumption decisions (Karaman & Bozok, 2023)​. Although algal proteins are promising regarding sustainability and nutritional value, barriers such as taste and aroma hinder consumer acceptance [15]. Furthermore, microbial-based foods require careful assessment due to potential risks associated with food safety, toxin formation, heavy metal accumulation, and allergic reactions [16]. Another alternative, cultured meat, demonstrates considerably lower environmental impact than conventional livestock production, while offering protein quality comparable to traditional meat [17]​. Nevertheless, realizing these advantages depends on production supported by renewable energy; otherwise, the high energy demand may result in a higher carbon footprint than expected [11]. Large-scale production, cost, and consumer acceptance remain ongoing challenges [11].

In this regard, edible insects emerge as a promising alternative food source, perceived as the “food of the future” due to their high protein content, low environmental footprint, and rapid reproduction capacity [9] ​. In 2013, FAO published the *Edible Insects: Future Prospects for Food and Feed Security*, highlighting their potential as food and feed, thereby sparking global interest in the field [18]. In Europe, the European Food Safety Authority (EFSA) has evaluated the safety of various insect species for human consumption, approving *Tenebrio molitor* (mealworm), *Acheta domesticus* (house cricket), and *Locusta migratoria* (migratory locust) in specific forms for human use [19]. These approvals have since been implemented through European Commission regulations [19]. In Asia, Singapore expanded its regulatory framework in 2023 by authorizing 16 insect species for human food use [20]. Today, more than 2,000 insect species are consumed globally [21, 22]​. Among the most widely consumed species are crickets (*Acheta domesticus*) and mealworms (*Tenebrio molitor*) [23, 24]. Existing research confirms these insects are rich in protein, iron, vitamin B12, high-quality fats, and dietary fiber [24–26]. Their amino acid profiles are comparable to those of conventional high-quality protein sources such as eggs and milk [25]. Furthermore, insects require substantially less water, feed, and land to produce the same amount of protein as traditional livestock [5, 27]​. They are also compatible with circular economy principles, as they can be fed with food waste and effectively used in the bioconversion of organic residues [21].

Consumer acceptance of edible insects varies depending on cultural context, knowledge level, presentation, and disgust or emotional responses [28, 29]​​. In Western societies, psychological barriers such as “disgust” and “perceived naturalness” are prominent [30]. Conversely, insect consumption is both traditional and part of daily diets in regions such as Africa, Asia, and Latin America [29]​. Studies aimed at enhancing consumer acceptance demonstrate that strategies such as information provision, tasting experiences, and processing methods that reduce insect visibility are effective [29, 31] ​. In addition, the food safety evaluation of insect-based proteins requires careful monitoring of microbiological risks, allergenic potential, and chemical contaminants (e.g., heavy metals) (EFSA, 2021; Poma et al., 2017)​​.

The primary aim of this review is to examine the role and potential of edible insects in the food industry within the framework of nutritional value, health safety, processing technologies, and consumer perception. The study comprehensively addresses nutrient composition (macro- and micronutrients, protein quality, etc.) and industrial processing steps (drying, extraction, fermentation, etc.) while highlighting practical applications through product examples. Furthermore, consumer attitudes, psychological barriers, and ethical-cultural factors shaping dietary behavior are assessed within the context of current models (PDT). In this respect, the study aims to provide a holistic contribution to the literature by positioning edible insects as a protein source and a crucial component of sustainable food systems.

## Some Edible Insect Species and Their Cultural Use

The consumption of edible insects is practiced by approximately 2 billion people worldwide [32]. In Southeast Asia, particularly in Thailand, insects such as silkworm pupae (*Bombyx mori*), bamboo caterpillars (*Omphisa fuscidentalis*), crickets (*Acheta domesticus*), weaver ants (*Oecophylla smaragdina*), and giant water bugs (*Lethocerus indicus*) are traditionally consumed [33]. In North America, particularly in Mexico, insects such as escamoles (ant larvae) and chapulines (grasshoppers) are part of the traditional cuisine [34, 35]. The European Food Safety Authority (EFSA) has evaluated the safety of various insect species within this context and has issued positive opinions for some species. The primary rationale for this limitation is that such processing methods significantly reduce microbial load (e.g., through boiling, drying, or freezing), stabilize nutritional components into identifiable states, and enable a more controlled assessment of potential risks such as allergenicity, heavy metals, and contaminants associated with different product forms [19].

The most commonly utilized insect species in the food industry are *Tenebrio molitor* (mealworm), *Acheta domesticus* (house cricket), and *Hermetia illucens* (black soldier fly larvae). These species are favored due to both their nutritional value and ease of production [36]. In 2021, EFSA published a scientific opinion confirming the safety of dried *Tenebrio molitor* larvae for human consumption [19]. Following this assessment, the European Commission authorized its commercialization as a novel food under Implementing Regulation (EU) 2021/882 [37]. Similarly, frozen, dried, and powdered forms of *Acheta domesticus* were evaluated by EFSA and subsequently approved as novel foods under Implementing Regulation (EU) 2022/188 [38].

## Industrial Production and Processing of Edible Insects

**Fig. 1:**
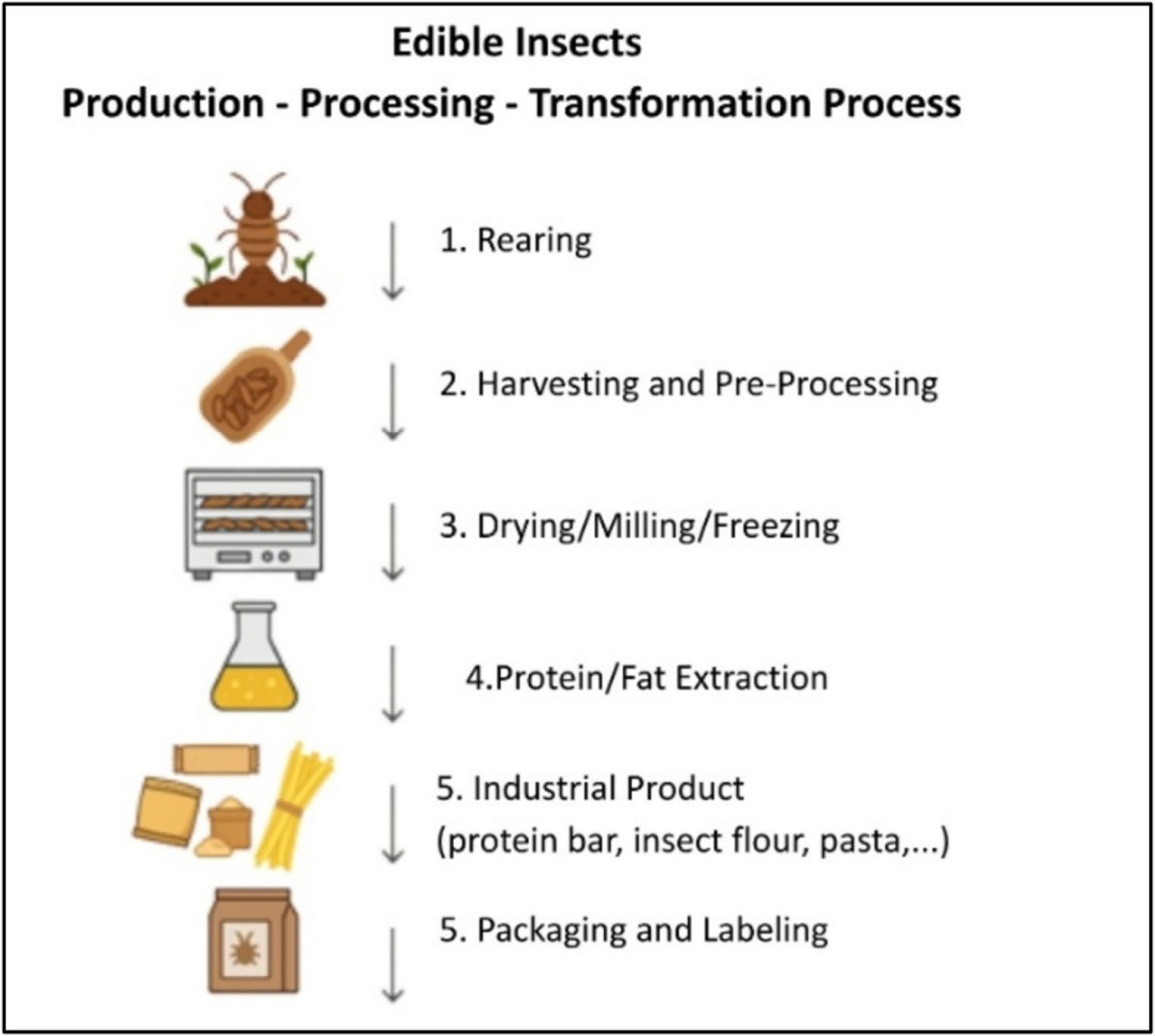
Production–processing–transformation process of edible insects.

As illustrated in Fig. 1, the process includes rearing, harvesting and pre-processing, drying/milling/freezing, protein and fat extraction, development of industrial products (protein bars, insect flour, pasta), and the final stage of packaging and labeling.

## Rearing Stages

Insect production is carried out under optimized conditions regarding temperature, humidity, and feeding regimen [39]. The rearing conditions of *Tenebrio molitor* (mealworm), *Acheta domesticus* (house cricket), and *Hermetia illucens* (black soldier fly) are presented in Table 1.

**Table 1:** Rearing conditions of *Tenebrio molitor* (mealworm), *Acheta domesticus* (house cricket), and *Hermetia illucens* (black soldier fly)

Species	Optimum Temperature (°C)	Humidity (%)	Feed Source	Life Cycle Duration	Oviposition Environment	Harvest Time
*Tenebrio molitor* (Mealworm)	25–28 [40]	60–70 [40]	Wheat bran, oat flakes, potato peels [41]	8–10 weeks [42]	Deposited on substrate surface [42]	Before pupation, when larvae reach ~ 170 mg [42]
*Acheta domesticus* (House Cricket)	27–30 [43]	60–70 [43, 44]	Corn, wheat bran, vegetable residues [43, 44]	3–4 months [43]	Moist peat or sand [42]	After the nymph stage, at adulthood [42]
*Hermetia illucens* (Black Soldier Fly)	32–35 [45]	60–70 [42, 45]	Fruit–vegetable pulp, kitchen waste, manure [45]	18–36 days [45]	500–900 egg clusters [46]	Prepupal stage [45, 47]

### *Tenebrio molitor* (Mealworm)

*Tenebrio molitor* is a terrestrial larva and a holometabolous insect (undergoing complete metamorphosis), transitioning from egg to larva, pupa, and finally adult [21]. The duration of the larval stage before pupation varies depending on temperature, lasting approximately 4 to 6 months [27]. Optimal rearing conditions include a 25–28 °C temperature and a relative humidity of 60–70% [26, 48]. At lower temperatures, development slows but larval size may increase. Rearing is generally carried out on low-cost substrates such as wheat bran, oat flakes, and potato peels. The substrate’s moisture content is typically 10–12% [48]. Within about 8–10 weeks, larvae reach an average weight of 170 mg, which is considered the optimal harvest time for protein quality [26]. Prior to harvest, a 24–48-hour fasting period is applied to allow gut clearance [27]. Pupation occurs when larvae are left in a dry environment; adults emerging from pupae reproduce, lay eggs, and the cycle restarts [27].

### *Acheta domesticus* (House Cricket)

*Acheta domesticus* completes its development from egg to adult within 6–8 weeks (Mancini et al., 2019; Morales-Ramos et al., 2018), and is an exopterygote species (undergoing incomplete metamorphosis) [44, 49]. Its average life cycle spans 3–4 months, most of which is spent in the nymph stage, during which individuals grow by successive molts. Nymphs are wingless and reproductively immature; however, this stage is crucial for commercial production due to rapid growth and high nutrient content [50]. The optimal rearing temperature is 27–30 °C with 60–70% relative humidity [18]. Lower temperatures prolong development and increase mortality rates. Diets usually consist of corn, wheat bran, and vegetable residues, with protein-enriched feeds (18–24% protein) used to improve yield [51]. Density management is critical in mass rearing, as overcrowding can trigger cannibalism and stress behaviors [51, 52]. Oviposition is facilitated using plastic containers filled with moist sand or peat, which are collected daily and transferred to incubation units [51].

### *Hermetia illucens* (Black Soldier Fly Larvae)

The black soldier fly can develop from egg to adult in approximately 14–20 days [53, 54]. The larval stage typically lasts 18–36 days, after which individuals enter the prepupal stage [55]. Optimal development occurs at 32–35 °C and 60–70% relative humidity [53]. Rearing substrates are usually enriched with organic waste such as fruit–vegetable pulp, kitchen waste, and manure, which provide ideal conditions for growth [53]. Females lay egg clusters of 500–900 eggs in cracks or porous structures near compost surfaces [56]. Once larvae reach maximum size, they transition into the prepupal stage, exhibiting migratory behavior, at which point they are harvested. Prepupae can either be directly processed or retained as breeding stock [21, 53].

The industrial-scale rearing of insects requires controlled climate conditions, automated feeding systems, and strict hygiene protocols [18]. However, these production systems still face regulatory gaps, high initial investment costs, and limited consumer acceptance [50]. In recent years, technological innovations such as robotic harvesting systems and IoT-based monitoring tools have been developed and implemented to reduce manual labor and enhance production efficiency [57, 58].

## Comparison of the Advantages and Disadvantages of Insect Processing Technologies

The most commonly used methods in processing edible insects include drying, grinding, extrusion, and fermentation [39]. Drying reduces the moisture content of the product, prevents microbial spoilage, and extends shelf life; this process can be carried out through different technologies such as hot-air drying, freeze-drying, and steam treatment [21]. Once dried, insects are ground into flour and further processed in various food products [59]. Extrusion technology transforms insect proteins into a form that can be incorporated into convenience foods, pasta, and meat analogues [60, 61]. Conversely, fermentation is a biotechnological method applied to enhance both nutritional value and functional properties [62]. Various industrial processing methods applied to edible insects are presented in Table 2.

**Table 2:** Industrial processing methods applied to edible insects

Processing Method	Technique	Advantages	Disadvantages	References
Drying	Reduction of moisture content using hot air, vacuum, freeze-drying, or similar methods	- Prevents microbial spoilage - Extends shelf life and facilitates transport	- Prolonged process at low temperatures- Possible nutrient loss- High energy cost	[18, 39]
Steaming	Exposure of insects to high temperature without drying	- Preserves nutrients - Reduces allergenicity - Inactivates pathogens	- High water content may cause rapid spoilage- Possible loss of texture and softness	[63]
Grinding	Conversion of dried insects into powder (insect flour) using mechanical methods	- Easy integration into bakery products - High protein availability	- Defatting required for high-fat species- Possible undesirable taste and odor	[54]
Extrusion	High temperature and pressure treatment for textured product production	- Produces meat-like structure - Applicable in snacks, pasta, meat analogues	- High energy consumption- Risk of protein denaturation	[64]
Fermentation	Bioprocess using lactic acid bacteria or yeasts	- Improves digestibility - Enhances flavor - Reduces allergenicity	- Requires microbial control - Limited chitin degradation - Flavor profile may not be suitable for all products	[59, 62]
Alkaline Extraction & Isoelectric Precipitation	Solubilization of proteins through pH adjustment and precipitation	- Effective protein purification - Allows concentrated protein recovery	- High pH may denature proteins - Risk of chemical residues	[65]
Enzymatic Hydrolysis	Decomposition of proteins into small peptides using protease enzymes	- Improves digestibility - Enhances functional properties - Reduces allergenicity	- Costly process - Requires appropriate enzyme selection	[54]
Ultrasound Extraction	Disruption of insect tissues using high-frequency sound waves	- Reduces extraction stress - Shortens processing time - Improves solubility	- High equipment cost- Difficult scale-up	[66]
Supercritical CO₂ Extraction	Protein/lipid extraction using CO₂ in supercritical state	- Produces solvent free extracts - Effective fat removal	- High equipment cost- Slow extraction rate	[67]
Microwave-Assisted Processing	Microwave heating for drying or extraction acceleration	- Rapid process - Energy-efficient	- Risk of uneven heating - Possible reduction in protein quality	[68]

### Insect Flour Production

Insect flour is the most common processed derivative of edible insects, particularly obtained from species such as *Tenebrio molitor* and *Acheta domesticus* [39]. The production process generally begins with drying the insects by heat or freeze-drying, followed by grinding [69].

#### Drying

Drying reduces the moisture content of insects, thereby extending shelf life and preventing microbial growth [69]. Common methods include hot air oven drying, freeze-drying (lyophilization), and microwave drying [39]. Oven drying is usually performed at 60–80 °C for 4–8 h and is particularly suitable for species with high moisture content, such as *Hermetia illucens* [39]. Freeze-drying, although longer and performed at − 20 °C or lower, provides better preservation of nutrients compared to oven drying [69]. This process is mainly applied to *Tenebrio molitor* and *Acheta domesticus* to reduce microbial load and prepare them for subsequent processing [39]. Freezing largely preserves protein structures and minimizes cell wall disruption, thus stabilizing nutritional value [69]. However, during thawing, water loss and enzymatic activity may lead to quality reduction [69]. While energy consumption is costly in the long term, drying ensures microbial safety [39].

#### Steaming

Steaming is usually performed at 100 °C for 5–10 min, effectively eliminating pathogens and improving digestibility [39]. This method enhances protein digestibility while facilitating the breakdown of chitin, a poorly digestible structure [18]. *Tenebrio molitor larvae*,* once steamed and then dried*,* can be ground into flour*,* ensuring microbial stability of the product* [69]. From a consumer safety perspective, steaming is among the initial thermal treatments recommended in European Union regulations [18].

#### Grinding

Grinding is essential to obtain insect flour at the desired particle size without compromising structural integrity. The resulting flour is sieved to achieve a homogeneous structure [39]. In some applications, defatting is performed to produce a more stable and protein-rich flour [70]. This can be achieved through mechanical pressing or solvent extraction. Defatting not only extends the shelf life of the flour but also helps achieve the desired nutritional profile [70, 71].

### Lipid Extraction

Edible insects, particularly *Hermetia illucens* and *Tenebrio molitor*, contain 15–35% lipids [53]. These lipids are nutritionally valuable (high in linoleic and oleic acids) and can also serve functional purposes [71]. During protein isolation, the first step is defatting, achieved through mechanical pressing or solvent extraction.

#### Mechanical Pressing

Mechanical pressing involves compressing insects using screw or hydraulic presses to extract lipids [72] physically. Before pressing, insects are usually dried and ground to enhance extraction efficiency [73]. Once placed in the press, insect cell walls are ruptured, releasing lipids [74]. The extracted oil is collected, while the remaining press cake is protein-rich and suitable for further processing [73]. Mechanical pressing is environmentally friendly since it avoids chemical solvents. However, lipid yield is lower than solvent extraction, and the press cake may still contain residual oil, posing challenges for protein isolation [74].

#### Solvent Extraction

Solvent extraction is a method that employs organic solvents to remove lipids from insects. This process effectively eliminates residual lipids after mechanical pressing [73]. Commonly, organic solvents such as hexane are used, with the choice of solvent depending on the lipids’ solubility and the process’s safety [74]. The press cake is mixed with the selected solvent and incubated for a defined period, during which the lipids dissolve into the solvent [73]. The lipid–solvent mixture is then separated via distillation or evaporation, allowing the solvent to be recovered and the oil to be obtained [74]. The defatted cake from solvent extraction is rich in protein and suitable for protein isolation [73]. Solvent extraction provides high lipid recovery efficiency and is highly effective in removing residual fats after pressing. However, residual solvents may pose food safety concerns, while solvent recovery and disposal introduce additional costs and environmental burdens [74]. Mechanical pressing and solvent extraction are often applied sequentially: first, mechanical pressing removes the majority of the oil, and then solvent extraction eliminates the remaining lipids. This combination ensures both high efficiency and environmental sustainability [73]. The extracted insect oils find applications in food production and the cosmetics and biofuel industries [53].

### Protein Isolation

Proteins derived from edible insects offer high bioavailability compared to plant- and animal-based protein alternatives [69]. The lipid fraction is first removed in protein isolation, followed by protein purification using alkaline extraction and isoelectric precipitation techniques [51]. Some studies have reported that enzymatic hydrolysis can be applied to obtain functional proteins with enhanced emulsifying and foaming capacities [39]. These proteins are increasingly being used as functional food ingredients in various applications, from vegan dietary supplements to sports drinks [51].

#### Alkaline Extraction

**Fig. 2:**
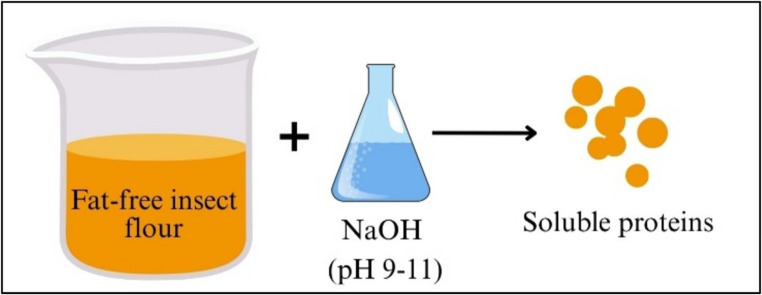
Isolation of protein from insect flour using the alkaline extraction method

As illustrated in Fig. 2, proteins are solubilized by treating defatted insect flour with NaOH solution (pH 9–11), resulting in the release of soluble proteins.

Alkaline extraction is applied to solubilize proteins from defatted insect flour. In this process, alkaline solutions such as sodium hydroxide (NaOH) with a pH range of 9–11 are typically used [52]. The alkaline environment increases protein solubility and thereby enhances extraction efficiency. However, since high pH values may cause protein denaturation, process conditions must be carefully controlled [65].

#### Isoelectric Precipitation and Enzymatic Hydrolysis

**Fig. 3:**
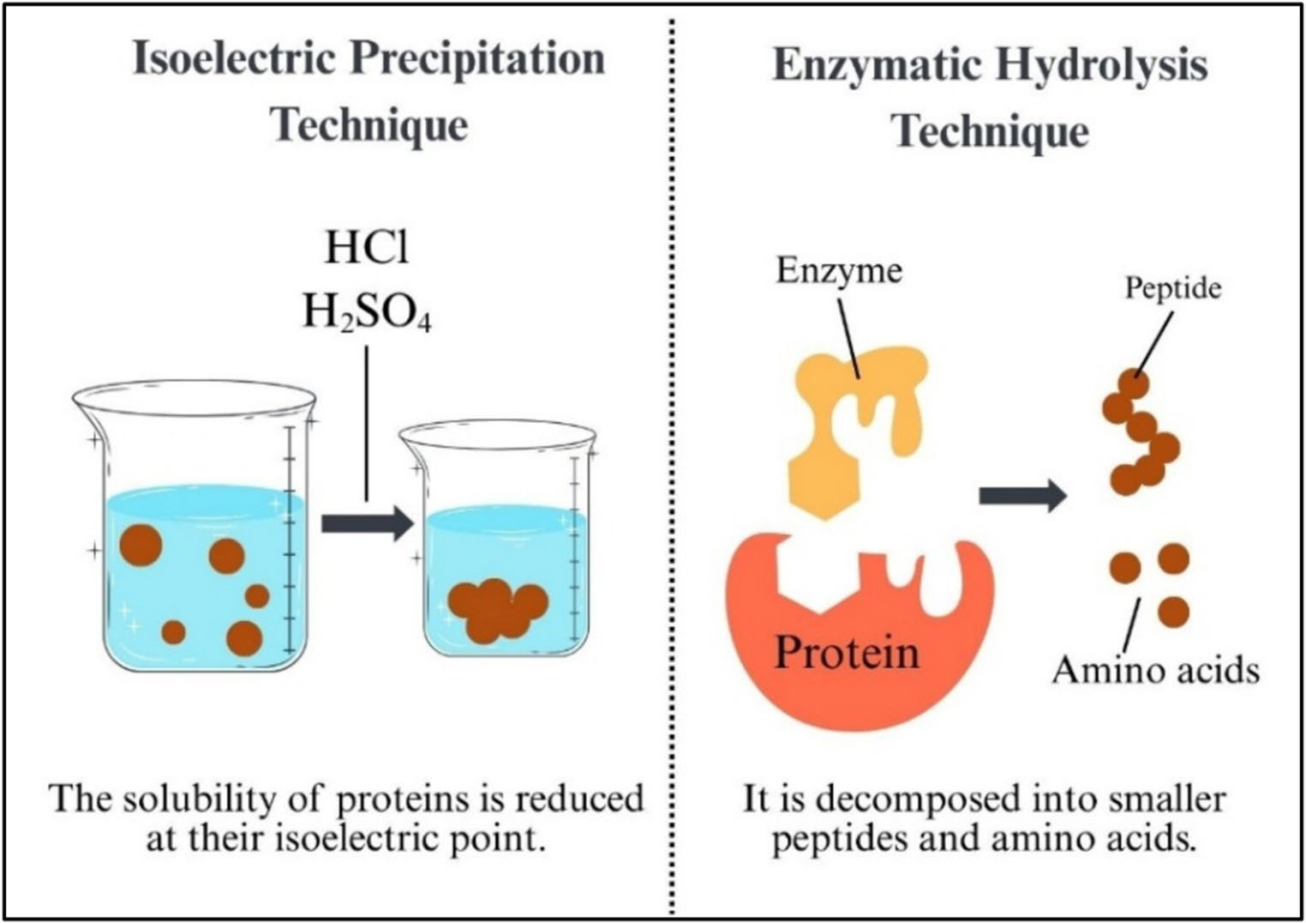
Isoelectric precipitation and enzymatic hydrolysis in the production of isolated protein

As illustrated in Fig. 3, insect protein isolation can be achieved through two main approaches: isoelectric precipitation and enzymatic hydrolysis.

Isoelectric precipitation is based on the principle that the solubility of proteins decreases to a minimum at their isoelectric point (pI), where their net electrical charge is zero. The pH value is usually adjusted to the pI of the proteins by adding hydrochloric acid (HCl) or sulfuric acid (H₂SO₄), which leads to protein precipitation [39]. This method is a practical step in protein purification. The purified proteins can subsequently be hydrolyzed into smaller peptides and amino acids through enzymatic hydrolysis. This process is carried out using protease enzymes to enhance the functional properties of proteins [54]. Enzymatic hydrolysis is favored in the food industry to improve protein digestibility and reduce allergenic potential [75].

### Fermentation

Fermentation is a traditional yet effective biotechnological method to enhance edible insects’ nutritional value and microbial safety [62]. This process is typically performed after pretreatments such as cooking or grinding, using lactic acid bacteria (e.g., *Lactobacillus plantarum*, *L. casei*) or yeast strains under controlled conditions [59]. Fermentation improves the digestibility of insect proteins while contributing to the reduction of antinutritional compounds and potential pathogens [49]. Additionally, this process can enhance sensory properties such as flavor and aroma, extend shelf life, and provide specific probiotic effects. However, not all fermentation processes yield the desired outcomes; if environmental conditions are not properly managed, spoilage risk increases, and undesirable odors may develop [59]. Furthermore, structural components such as chitin are not completely degraded during fermentation, and digestibility challenges may persist [59]. Recent studies indicate that fermentation can improve microbiological safety and consumer acceptance, offering a significant advantage for insect-derived ingredients integrated into functional foods [49].

## Nutritional Value and Bioavailability of Edible Insects

Edible insects possess an essential amino acid profile comparable to that of animal proteins, and the nutritional value of insect proteins has been the subject of intensive research in recent years [76]. The average contents of protein, fat, iron, omega-3, and omega-6 in insects and conventional animal protein sources (beef, chicken, fish) are presented in Table 3.

**Table 3:** Average protein, fat, iron, omega-3, omega-6 contents of insects and conventional animal protein sources (beef, chicken, fish)

Nutrient	Insects	Beef	Chicken	Fish	References
Protein (% dry)	35–61%	23–25%	23–25%	20–25%	[18, 26, 77]
Fat (% dry)	13–33%	15–20%	10–15%	5–10%	[26]
Iron (mg/100 g)	8–20 mg	~ 6 mg	~ 1 mg	~ 1 mg	[36, 78]
Omega-3 (g/100 g)	0.1–0.5 g	~ 0.02 g	~ 0.05 g	0.5–1.5 g	[26]
Omega-6 (g/100 g)	0.5–2.0 g	0.5–1.0 g	1.0–2.0 g	0.1–0.5 g	[26, 79]

Edible insects possess a high protein content, ranging from 35% to 61% on a dry weight basis, depending on the species [18]. Insect proteins contain all essential amino acids; however, in certain species, the levels of amino acids such as methionine and tryptophan may be limited [52]. *Tenebrio molitor* (yellow mealworm) and *Acheta domesticus* (house cricket) generally meet the WHO/FAO requirements for amino acid balance. In contrast, *Locusta migratoria* (migratory locust) has been reported to possess borderline levels of tryptophan [32]. One of the most commonly used methods for assessing protein quality is the Protein Digestibility Corrected Amino Acid Score (PDCAAS), which reflects both digestibility and amino acid composition [80]. While conventional animal protein sources and insect species generally exhibit high PDCAAS values (e.g., 1.0 for egg, 0.86 for mealworm), plant-based proteins often show lower values (e.g., 0.39–0.54 for wheat) [81]. Thus, insect proteins can present higher PDCAAS values than some plant-based protein sources, though they are usually slightly lower than animal proteins. Nevertheless, the amino acid profiles of insect proteins are particularly rich in essential amino acids, making them a valuable alternative for human nutrition [52]. owever, PDCAAS has certain limitations, particularly its truncation of values at 1.0 and its inability to fully reflect the digestibility of individual indispensable amino acids [18]. To address these limitations, the Digestible Indispensable Amino Acid Score (DIAAS) has been proposed as a more precise indicator of protein quality [82]. Unlike PDCAAS, DIAAS evaluates the digestibility of individual indispensable amino acids at the ileal level rather than using total fecal digestibility, thereby providing a more accurate estimation of amino acid bioavailability [18, 82]. Recent studies suggest that several edible insect species demonstrate favorable DIAAS values comparable to high-quality animal protein sources [83]. Nevertheless, the digestibility of insect proteins may vary depending on several factors. These include the insect species, processing conditions such as drying, defatting, or enzymatic hydrolysis, and the presence of chitin in the insect exoskeleton [83, 84]. Chitin may reduce protein digestibility by limiting enzyme accessibility during digestion; however, certain processing techniques can partially mitigate this effect and improve amino acid availability [84]. Therefore, both biological characteristics and technological processing steps should be considered when evaluating the nutritional quality of insect-derived proteins.

The digestibility-based quality assessment of selected edible insect species and various animal- and plant-based protein sources in terms of PDCAAS and DIAAS scores is presented in Table 4. These protein sources exhibit variability in PDCAAS and DIAAS values, reflecting the diversity in their amino acid profiles and digestibility [52].

**Table 4:** Digestibility-based quality assessment (PDCAAS & DIAAS) of selected edible insect species and various animal- and plant-based protein sources

Protein Source	PDCAAS (%)	DIAAS (%)
*Hermetia illucens* (black soldier fly larvae)	~ 78 [83]	57 [83]
*Acheta domesticus* (house cricket)	83 [83]	76 (in vivo) [83]
*Tenebrio molitor* (mealworm)	76 [83]	54 (in vivo) [83]
Beef	92 [85, 86]	111–116 [87]
Chicken breast	95 [88]	108 [89]
Fish (e.g., Tuna)	100 [88]	—
Egg	100 [85]	101 [90]
Cow’s milk protein (whey)	100 [85]	90–100 [82]
Cow’s milk protein (casein)	100 (1.31) [85, 88]	118 [82]
Soy protein isolate	98 [91]	89 (in vitro) [82]
Peanut protein	78 [87]	64 (in vitro) [87]
Chickpea protein	74 [92]	83 (in vitro) [82]
Wheat protein	~ 45 [86, 87]	40–48 (in vitro) [87]

In addition to protein and amino acid content, insects contain between 13% and 33% fat, rich in unsaturated fatty acids [26]. The fatty acid profile of insects may vary depending on their diet. For instance, supplementing the diet of house crickets and mealworms with 2% flaxseed oil has been shown to increase their omega-3 fatty acid content and reduce the omega-6/omega-3 ratio to below 5, which is considered optimal for human health [79]. Regarding fiber, insects contain chitin, a type of dietary fiber located in their exoskeleton, and the amount of chitin varies by species [78]. Some studies have demonstrated that chitinase enzymes in the human gut microbiota can partially degrade chitin [93]. Moreover, reducing the chitin content may improve the digestibility of insect proteins [78]. Regarding micronutrients, insects are a valuable source of essential vitamins and minerals, including iron, zinc, calcium, and vitamin B12 [36].

## Integration of Edible Insects into Food Products

After undergoing specific pre-treatment processes and conditions (e.g., washing, drying), insects can be processed into various products such as flour, protein isolates, and extracted oils, which are then incorporated into food items [26]. These insect-derived products are commonly added to foods such as protein bars, pasta, bread, and crackers at 10% to 30%, enhancing their nutritional value [71]. In particular, bakery products fortified with 10–20% cricket flour have been reported to improve protein quality while maintaining acceptable sensory attributes [59]. Table 5 presents the formulation, processing steps, and sensory effects of selected industrial food products produced with insect flour.

**Table 5:** Formulation, processing steps, and selected sensory effects of industrial food products produced with insect flour

Product Type	Formulation	Processing Steps	Sensory Effects	References (APA)
Protein Bar	Insect flour, oats, hazelnut paste, honey	Mixed, shaped, baked or cooled and frozen	Portability, nutty flavor	[36, 84, 94]
Pasta	5–30% insect flour + semolina/egg to balance the formula	Dough prepared, extruded, dried	Nutty taste, darker color	[54, 70]
Bread	5–15% insect flour + wheat flour/gluten supplementation may be required	Fermented, baked, color and flavor control applied	Distinct flavor	[49]
Crackers	10–20% insect flour / additional fat may be needed	Dough rolled out, shaped, baked at 170–180 °C	Increased crispiness	[95]

### Protein Bars

In protein bar production, insect flour is used as a high-quality protein source. It is usually blended with binding agents (such as date paste or honey) and cereal-based ingredients to optimize nutritional content and structural properties [94]. Bars supplemented with 15–30% insect flour have been reported to exhibit increased protein and fiber content, while maintaining acceptable levels of consumer sensory approval [96]. A study conducted in Poland demonstrated that including mealworm, cricket, and buffalo worm flours at levels of 15% and 30% significantly enhanced the protein and fiber contents of the bars [96]. In the same study, bars containing 15% buffalo worm flour received the highest consumer acceptance scores. Enzymatic pre-treatments have shown potential to improve both nutrient bioavailability and flavor profiles, while extending these products’ shelf life [96]. Protein bar formulations are processed either by low-temperature baking or by cooling to achieve structural stability, during which insect flour’s adhesive and texturizing properties are utilized [36]. Before packaging, microbiological safety checks are performed, and nutritional labeling is completed [22, 36].

### Bakery Products

Edible insect flours, particularly rich in protein, healthy fats, and dietary fiber, are increasingly used to enhance the nutritional value of bakery products such as bread, pasta, and crackers [97]. In pasta production, insect flour is incorporated at 5–30% levels as a partial replacement for traditional semolina, thereby improving protein content and amino acid balance [54]. However, higher inclusion rates can negatively affect sensory properties such as taste, aroma, and texture; thus, 10–15% incorporation levels are generally recommended [97]. During dough formulation, insect flour contributes to consistency but may reduce elasticity due to its lack of gluten. It is commonly balanced with egg addition or mixed with gluten-containing flours [70]. Pasta processed through conventional extrusion and drying develops a darker brown hue and a slightly nutty flavor profile when enriched with insect flour [39]. In bread formulations, insect flour is typically incorporated at 5–15% substitution levels [49, 59]. Since insect flour lacks gluten, inclusion rates above 10% are often limited to prevent weakening of the gluten network [98]. Furthermore, the fermentation process requires careful adjustment, as the high lysine content of insect flour can accelerate fermentation and result in darker crust coloration [94]. Post-baking analyses have demonstrated significant increases in iron and zinc contents in breads fortified with insect flour [99]. In crackers, insect flour is generally used at substitution levels of 10–20% [23]. The dough is rolled, shaped, and baked at 170–180 °C for 10–15 min, yielding products with superior nutritional profiles compared to conventional crackers [49]. Consumer tests have shown that adding flavoring strategies, such as incorporating spices or garlic, enhances acceptance [94]. Sensory evaluations consistently report that products with up to 10% insect flour are rated at similar acceptance levels to control samples [100].

In addition to insect flours, extracted insect oils are gaining increasing attention in the food industry. Oils derived from species such as black soldier fly larvae *(Hermetia illucens)* and mealworms *(Tenebrio molitor)* can partially or fully replace conventional fats in bakery products including margarine, biscuits, and cakes [101]. For example, substituting 25–50% of butter with *Hermetia illucens* larval oil in cakes and waffles has yielded products with acceptable sensory properties [77].

### Functional and Industrial Integration

Incorporating insect-derived products into foods holds potential not only as a protein source but also as functional additives [39]. *Tenebrio molitor* flour, due to its lack of gluten, may serve as an alternative ingredient for individuals with celiac disease, while chitin and its derivatives exhibit potential prebiotic effects [18]. Moreover, these components can perform technological functions such as emulsifiers, binders, and stabilizers [51].

### Health-Related Food Safety Profile

The food safety risk analysis for edible insects is presented in Table 6.

**Table 6:** Food Safety Risk Analysis for Edible Insects

Type of Hazard	Possible Risks	Explanation
Microbiological	- *Salmonella spp.* - *Campylobacter spp.* - *Bacillus cereus*	Contamination under inadequate production–processing conditions [18, 102].
Chemical/Allergenic	- *Listeria monocytogenes* - Heavy metals (lead, arsenic, cadmium, mercury) - Pesticide residues - Environmental contaminants - Allergenic proteins: tropomyosin, arginine kinase	May occur depending on rearing environment and substrates. Individuals with shellfish allergies are at particular risk [16, 102, 103].
Physical	- Hair, sand, plastic fragments - Metal particles from processing equipment - Insect exoskeleton residues (excess chitin)	Physical hazards may arise due to inadequate cleaning, sorting, or equipment maintenance [18].

While edible insects stand out as a sustainable and nutritious protein source, their safety from a health perspective requires careful management of microbiological, chemical/allergenic, and physical risks [19]. This can be achieved through proper rearing, processing, and labeling practices [18].

When reared or processed under inadequate conditions, edible insects can become contaminated with pathogenic bacteria such as *Salmonella*,* Campylobacter*, and *Bacillus cereus* [102, 104]. These microorganisms may originate from insects’ feeding substrates or environmental factors [19]. However, it has been shown that proper thermal treatments (e.g., frying, boiling, drying) can effectively eliminate these pathogens [39].

From a chemical perspective, edible insects can accumulate heavy metals (arsenic, lead, cadmium, mercury), pesticide residues, and other environmental contaminants depending on their surrounding environment [16]. This risk is notably higher in insects collected from the wild [105]. Therefore, strict monitoring and control during rearing and processing are necessary to ensure their safe consumption [19]. At the same time, edible insects may trigger cross-reactive allergic reactions, especially in individuals allergic to shellfish (e.g., shrimp, crab), due to proteins such as tropomyosin and arginine kinase [103]. This cross-reactivity arises from the similarity of protein structures between insects and shellfish (Verhoeckx et al., 2016). For this reason, allergen warnings should be clearly stated when labeling insect-based products [19]. The processing methods applied to insects are critical in reducing microbiological and chemical risks [39]. For instance, drying, frying, and extraction can inactivate pathogens and help remove toxic compounds [18]. Moreover, although these processes may not directly eliminate allergens, they are known to alter the structure of allergenic proteins [106].

## Environmental Sustainability and Economic Feasibility

Conventional meat production is a significant source of greenhouse gas emissions. According to LCA (Life Cycle Assessment) studies conducted by Poore and Nemecek (2018), beef production results in approximately 60 kg of CO₂ equivalent emissions per kilogram of meat [5]. Cultured meat can reduce this value by up to 80% in the best-case scenarios; however, if the energy source is not renewable, its carbon footprint advantage remains limited [107]. On the other hand, plant-based and insect proteins rank among the most environmentally friendly alternatives in terms of total carbon footprint during production and processing [108].

Insect-based protein production requires significantly less water, feed, and land use per unit than animal protein and cultured meat, lowering overall production costs [27]. For example, the feed conversion ratio in *Tenebrio molitor* production is approximately 1.5:1, whereas this ratio is 8:1 for cattle [21]. This makes insect protein more economically attractive. In addition, insects’ short life cycles and suitability for vertical farming systems help reduce both land requirements and labor needs [53]. In microbial and insect-based systems, agricultural waste can be directly used as a nutrient substrate, reducing waste and generating valuable protein products [109]. Specifically, black soldier fly larvae can efficiently convert food waste while contributing to farming systems through organic fertilizer production [110]. The expansion of such systems enhances food security and serves as a model for integrated farming [22].

The techniques used in insect protein isolation (e.g., alkaline extraction, isoelectric precipitation, enzymatic hydrolysis) impose different economic burdens depending on energy and enzyme costs [39]. Particularly, high value-added processes like enzymatic hydrolysis may pose cost barriers for small-scale enterprises. However, when these processes are scaled up, unit costs decrease significantly [54]. High-tech methods such as freeze-drying and supercritical CO₂ extraction may require high initial investments, but they provide returns in the long run due to improved efficiency and product quality [71].

The economic success of insect-based products depends on production costs and market acceptance [111]. Studies conducted in Western countries revealed that the “disgust factor” is high, yet consumption is more acceptable when products are processed into unrecognizable forms [27, 31, 111]. Similarly, an analysis conducted in Europe revealed that consumers are more likely to accept insect-based products in powdered, processed forms [94]. This trend increases demand for integrated products such as isolated proteins and improves marketability. Nevertheless, factors such as consumer trust, legal regulations, and food habits still pose barriers to economic feasibility [50].

## Consumer Perception and Behavioral Barriers

Studies on alternative protein consumption have shown that attitudes are generally the strongest determinant, followed by perceived behavioral control [10, 112] ​. For example, information highlighting the low environmental impact of products or their contribution to animal welfare helps individuals develop positive attitudes, positively influencing consumption intentions [113]​. In contrast, subjective norms may be less effective, particularly among individuals with weak family or peer influence, reflecting the balance between personal choices and social pressures.

Alternative proteins, particularly insect-based and lab-grown meat products, are the categories that face the most significant resistance due to neophobia [31]. Research indicates that food neophobia is influenced by age, education level, and cultural background, with resistance to novel foods increasing [114]. In neophobic individuals, this resistance manifests through psychological reactions such as disgust, alienation, and fear, and is often reinforced by the belief that alternative foods pose health risks [31]. Consumers’ initial response to food is based mainly on sensory evaluation, showing that taste, smell, texture, and presentation play a significant role in accepting alternative proteins [2]. In particular, for meat-like products, when textural similarity to real meat is unsatisfactory, rejection rates increase [115]. The direct presentation of insects in whole form often triggers disgust; however, acceptance improves when offered in powdered form or incorporated into other foods [114]. Thus, the physical appearance and the presentation mode directly shape consumer perception [28]. Consumer behavior is shaped not only by physiology but also by ethical and cultural frameworks [116]. Certain animal species or production methods are prohibited in religions such as Islam, Judaism, and Hinduism, directly linking perceptions of alternative proteins with religious norms [111]. While insect consumption is traditionally common in some cultures, it is perceived as “dirty” and “unnatural” in Western societies, clearly reflecting the impact of cultural habits [21]. Consumer trust in alternative foods largely depends on perceptions of naturalness [117]. When perceived naturalness decreases, consumer trust declines and acceptance levels drop significantly [118]. For instance, lab-grown meat is frequently regarded as “artificial,” “processed,” or carrying “unknown risks” [108]. To enhance consumer trust, it is essential to provide transparent information about production processes and ensure scientific assurance of safety [31].

## Industrial Scalability Challenges

Despite the promising nutritional and environmental benefits of edible insects, several challenges remain regarding their large-scale industrial production. The successful scaling of insect-based food systems requires the standardization of farming conditions, including controlled temperature, humidity, feed composition, and hygienic rearing environments to ensure consistent product quality and microbiological safety [21, 110]. In addition, technological challenges associated with processing—such as efficient drying, lipid extraction, protein isolation, and the management of structural components like chitin—may influence both the economic feasibility and functional properties of insect-derived ingredients [109]. Furthermore, the expansion of the edible insect sector depends on regulatory harmonization, supply chain development, and improvements in consumer acceptance in Western markets, where entomophagy is still relatively unfamiliar [21, 49]. Addressing these technological, regulatory, and socio-cultural barriers will be essential for the sustainable integration of insect-based proteins into mainstream food systems.

## Conclusion

Edible insects are considered a nutrient-rich and environmentally sustainable food source due to their high protein bioavailability, fiber, vitamin (particularly B12), and microelement content. Their short life cycle, ability to thrive on minimal feed, and capacity to valorize organic waste align well with circular economy goals. However, health-related concerns such as microbiological risks (*Salmonella spp.*,* Bacillus spp.*), chemical contamination (pesticide residues, heavy metals), and allergenic potential (cross-reactivity with shellfish via tropomyosin) remain significant. In this context, the minimization of risks through processing techniques such as thermal treatment (frying, drying), extraction, and fermentation, as well as the adoption of Hazard Analysis and Critical Control Points (HACCP)-based hygiene practices throughout the production chain, is recommended. Furthermore, behavioral barriers such as disgust and food neophobia among consumers can be overcome through sensory testing, tasting experiences, and transparent labeling strategies.

Although numerous studies have explored the nutritional value, processability, and sustainability of edible insects, randomized controlled clinical trials on their long-term health impacts remain scarce. The risk of allergenicity—particularly related to proteins such as tropomyosin—is acknowledged; however, species-specific differences, structural modifications following thermal processing, and individual sensitivity levels require more detailed investigation. In addition, there is a need for ethnographic research to understand how local consumption practices in developing countries can be integrated into modern industrial applications and to identify culturally acceptable product formats. Despite the surge of academic interest following FAO’s 2013 report, the classification, ethical acceptance, and regulatory frameworks for edible insects as food remain unclear in many countries [18, 50]. In the European Union, in line with EFSA evaluations, several insect species *(Tenebrio molitor*,* Locusta migratoria*,* Acheta domesticus)* have been approved in specific forms as “Novel Foods”, granting consumption authorization [19]. However, legal uncertainty persists in many regions, lacking standards for labeling, allergen declaration, and assessment of animal-derived content. Moreover, in some communities, the ethical compatibility of insects with the definition of “clean food” is questioned, particularly within religious beliefs and vegan/vegetarian lifestyles.

Therefore, for insect-based foods to be adopted on a global scale, it is necessary to:

- Harmonize international regulations,
- Develop mandatory labeling requirements such as allergen declarations and sustainability claims,
- Strengthen education and information policies.

In conclusion, edible insects represent a valuable alternative for sustainable food systems across the nutrition, environmental, and economic dimensions. However, realizing this potential in a safe, culturally acceptable, and ethically grounded manner depends on the coordinated management of scientific research, regulatory systems, and consumer populations through an integrated strategy. 

## Key References

- Abro Z, Sibhatu KT, Fetene GM, Alemu MH, Tanga CM, Sevgan S, et al. (2025) Global review of consumer preferences and willingness to pay for edible insects and derived products. Global Food Security 44:100834. 10.1016/j.gfs.2025.100834.
  - ○ This large-scale global meta-analysis synthesizes data from over 50 studies on consumer acceptance of edible insects. It identifies major determinants of willingness to pay — including cultural familiarity, product form, and information exposure — and provides critical insights for designing consumer education and marketing strategies. The study highlights how socio-demographic and psychological factors shape acceptance, making it highly relevant for behavioral and market-level analyses in edible insect research.
- Copelotti E, Fratini F, Sforza G, Tuccinardi T, Demontis GC, Mancini S. (2025) Are Insect-Based Foods Healthy? An Evaluation of the Products Sold in European E-Commerce. Foods 14:1450. 10.3390/foods14091450.
  - ○ This recent cross-sectional study evaluates the nutritional composition, labeling practices, and safety claims of insect-based products available in European online markets. It provides up-to-date evidence on the macronutrient and micronutrient quality of these products and highlights discrepancies in regulatory labeling and allergen declarations. The findings directly inform both public health discussions and policy development regarding edible insect commercialization.

## Data Availability

No datasets were generated or analysed during the current study.
